# *De Novo* Genome Assembly of a Plant-Associated Rhodococcus qingshengii Strain (RL1) Isolated from Eruca sativa Mill. and Showing Plant Growth-Promoting Properties

**DOI:** 10.1128/MRA.01106-19

**Published:** 2019-11-14

**Authors:** Theresa Kuhl, Marius Felder, Thomas Nussbaumer, Doreen Fischer, Susanne Kublik, Soumitra Paul Chowdhury, Michael Schloter, Michael Rothballer

**Affiliations:** a Institute for Network Biology (INET), Helmholtz Center Munich, Neuherberg, Germany; b Research Unit for Plant Genome and Systems Biology (PGSB), Helmholtz Center Munich, Neuherberg, Germany; c Research Unit for Comparative Microbiome Analysis (COMI), Helmholtz Center Munich, Neuherberg, Germany; d Institute for Environmental Medicine (IEM), UNIKA-T, Augsburg, Germany; University of Maryland School of Medicine

## Abstract

Rhodococcus qingshengii RL1 was isolated from surface-sterilized leaves of Eruca sativa Mill. and shows plant growth-promoting (PGP) properties. The *de novo* genome assembly consists of one chromosome with 6,253,838 bp and two plasmids with 144,038 bp and 448,745 bp. Many genes could be identified reflecting its PGP potential.

## ANNOUNCEMENT

The genus *Rhodococcus* belongs to the phylum *Actinobacteria* and includes aerobic, Gram-positive, nonsporulating bacteria isolated from a broad variety of environments (1–3). Some of these bacteria have large genomes (>5 Mb) with high G+C content (1–4). Their ability to degrade a large spectrum of environmentally problematic compounds (2, 5) or perform quorum quenching (4) makes them suitable for bioremediation or agricultural applications.

RL1 was isolated from leaves of Eruca sativa Mill. Leaves were surface sterilized with 12% NaOCl, washed with sterile water, and macerated with sterile saline. The extract was plated on R2 agar and allowed to grow at 22°C for 5 days. Selected colonies were picked and allowed to grow on tryptic soy agar (TSA) and R2 agar at 28°C. For sequencing genomic DNA from RL1, a single colony picked from an agar plate was grown overnight in tryptic soy broth at 28°C. DNA was isolated via standard phenol-chloroform extraction with previous lysis with 600 μg/ml ampicillin for 3 h before extraction. For the PacBio Sequel system, the library was prepared with the SMRTbell template prep kit 1.0‐SPv3 and SMRTbell barcoded adapter complete prep kit-96. PacBio sequencing was performed with the Sequel sequencing kit 2.0 (8 reactions) and single-molecule real-time (SMRT) cell 1 M v2 tray. For Illumina MiSeq sequencing, the library was prepared using the TruSeq DNA PCR-free library preparation kit (Illumina, San Diego, CA, USA). Genomic DNA was fragmented by applying the Covaris E220 system according to the manufacturer’s protocol for a 550-bp average insert size and sequenced using MiSeq reagent kit v3 (600 cycles) (Illumina).

A total of 376,794 PacBio long reads (average read lengths of 15,245 bp, 16,813 bp, and 34,341 bp [3 SMRT cells]; 209× coverage) and a total of 1,068,580 Illumina short reads (read length, 300 bp; 49× coverage), quality checked with FastQC 0.11.8 (6), were included in the *de novo* assembly of the RL1 genome using the hybrid assembler MaSuRCA 3.2.1_01032017 (7). Sequence assembly produced three contigs representing one chromosome and two plasmids (chromosome, 6,253,838 bp; plasmid 1, 144,038 bp; plasmid 2, 448,745 bp) with a G+C content of 62.4%. The chromosome and plasmid 1 were circularized with Circlator version 1.5.5 (8). A total of 6,652 coding sequences were predicted with Rapid Annotations using Subsystems Technology (RAST) 2.0 (9), and gene clusters were identified with antiSMASH 4.2.0 (10) and Plant-bacteria Interaction Factors Resource (PIFAR) (11). All tools were used with their default settings. Many identified gene clusters were associated with traits relevant for (beneficial) microbe-plant interactions, including siderophore production, indole acetic acid (IAA) production, osmoregulation (ectoine), glucosinolate metabolism (β-glucosidase and *msrB*), quorum quenching (*qsdA*), antibiotic production, biofilm formation, lipopolysaccharide production, multidrug resistance, microbe-associated molecular patterns (MAMPs), heavy metal tolerance, and reactive oxygen species resistance.

**FIG 1:**
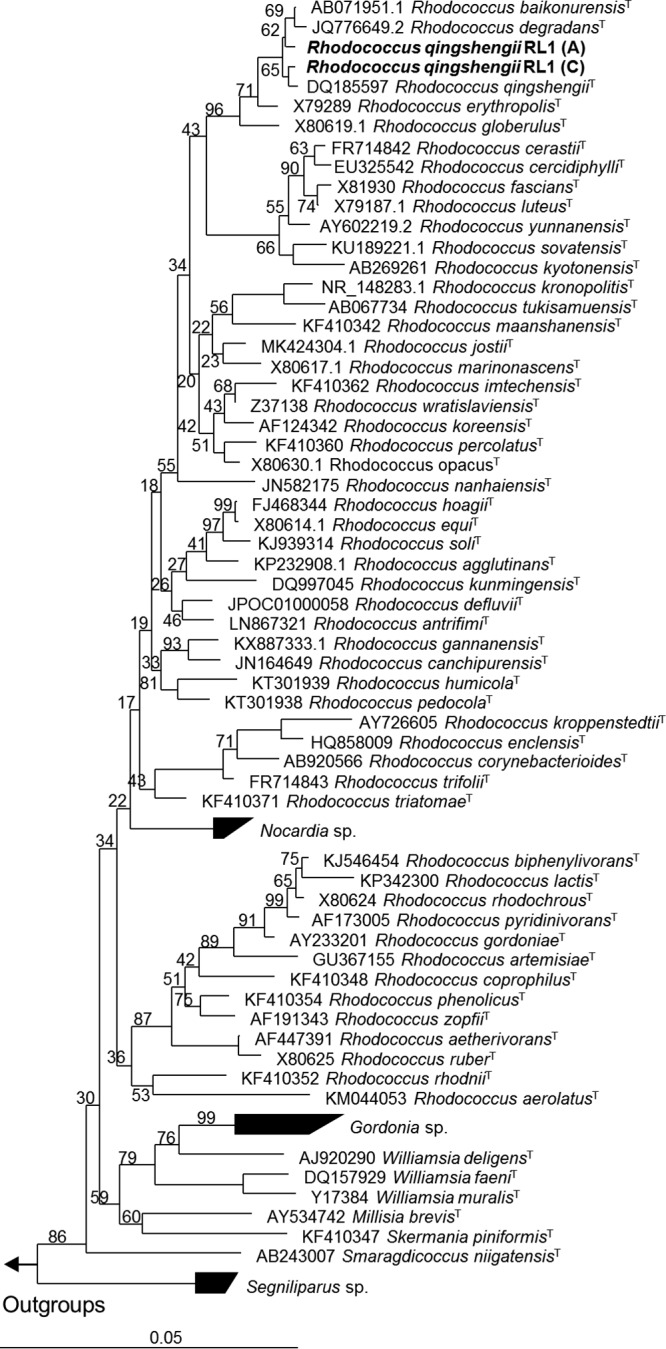
Neighbor-joining phylogenetic tree based on 16S rRNA sequences showing the phylogenetic relationship between Rhodococcus qingshengii RL1 and other members of the genus *Rhodococcus* and the family *Nocardiaceae*. Bootstrap values (%) for 1,000 resamplings are given at the nodes. Two versions of the 16S rRNA gene in the RL1 genome are included, differing at position 1074 (A or C) of the complete 16S rRNA gene.

The *de novo* assembly of the RL1 genome showed the highest similarity of >99% sequence identity with over 90% of the Rhodococcus qingshengii djl-6^T^ genome, and the 16S rRNA genes of RL1 were 99.9% identical to those of djl-6^T^. In a 16S rRNA gene-based phylogenetic neighbor-joining tree (12) calculated with ARB 5.3 (13), RL1 was placed within a cluster (bootstrap support, 99%) consisting of Rhodococcus erythropolis^T^, Rhodococcus qingshengii djl-6^T^, Rhodococcus degradans CCM 4446^T^, and Rhodococcus baikonurensis^T^ (Fig. 1). Further phylogenetic analysis of the *gyrB* gene verified the taxonomic classification of RL1 as Rhodococcus qingshengii (data not shown).

### Data availability.

This whole-genome sequencing project was deposited in GenBank under accession no. CP042915, CP042916, and CP042917 and in SRA (raw data) under accession no. SRR10070368 and SRR10070367.
